# Behaviour and Properties of Eco-Cement Pastes Elaborated with Recycled Concrete Powder from Construction and Demolition Wastes

**DOI:** 10.3390/ma14051299

**Published:** 2021-03-08

**Authors:** Laura Caneda-Martínez, Manuel Monasterio, Jaime Moreno-Juez, Sagrario Martínez-Ramírez, Rosario García, Moisés Frías

**Affiliations:** 1 Eduardo Torroja Institute for Construction Sciences (IETcc-CSIC), 28033 Madrid, Spain; laura.caneda@ietcc.csic.es (L.C.-M.); manuel.monasterio@ietcc.csic.es (M.M.); 2 Tecnalia, Basque Research and Technology Alliance (BRTA), Astondo Bidea, Edificio 700, Parque Tecnológico de Bizkaia, 48160 Derio, Spain; jaime.moreno@tecnalia.com; 3 Institute for the Structure of Matter (IEM-CSIC), 28006 Madrid, Spain; sagrario@iem.cfmac.csic.es; 4 Departamento de Geología y Geoquímica, Geomateriales Unidad Asociada CSIC-UAM, Universidad Autónoma de Madrid, 28049 Madrid, Spain; rosario.garcia@uam.es

**Keywords:** construction and demolition waste, supplementary cementitious materials, circular economy, eco-efficient cements

## Abstract

This work analyses the influence of fine concrete fractions (<5 mm) of different natures —calcareous (HcG) and siliceous (HsT)—obtained from construction and demolition waste (C&DW) on the behaviour of blended cement pastes with partial replacements between 5 and 10%. The two C&DW fractions were characterised by different instrumental techniques. Subsequently, their lime-fixing capacity and the physico-mechanical properties of the blended cement pastes were analysed. Lastly, the environmental benefits of reusing these fine wastes in the manufacture of future eco-efficient cement pastes were examined. The results show that HsT and HcG exhibit weak pozzolanic activity, owing to their low reactive silica and alumina content. Despite this, the new cement pastes meet the physical and mechanical requirements of the existing regulations for common cements. It should be highlighted that the blended cement pastes initially showed a coarser pore network, but then they underwent a refinement process between 2 and 28 days, along with a gain in compressive strength, possibly due to the double pozzolanic and filler effect of the wastes. The environmental viability of the blended cements was evaluated in a Life Cycle Assessment (LCA) concluding that the overall environmental impact could be reduced in the same proportion of the replacement rate. This is in line with the Circular Economy goals and the 2030 Agenda for Sustainable Development.

## 1. Introduction

Construction is a key sector for the progress of any modern society. In partiular, the development of cement-based materials—the most widespread in construction—has immensely facilitated the progress of humankind [1,2]. The main reason behind this success is the fact that the raw materials required for the manufacturing of cement and concrete are abundant, inexpensive and easily found around the world. As a consequence, the resources needed for construction have been generally treated as limitless, or at least this was the attitude up until recently.

The fact is, even though we still count on a wide reserve of raw materials to support the construction industry, the environmental impact of exploiting this kind of non-renewable resources cannot be ignored any longer. Such impacts include deforestation, soil erosion, air and water pollution and diversity loss in the affected areas [3,4]. The seriousness of the situation is aggravated by the large quantities of raw materials needed for construction. For instance, in 2018, 94% of the non-metallic minerals consumed in the European Union were destined for construction use, which amounted to 44% of the total raw materials demanded by the State Members [5].

Nevertheless, the lack of sustainability of the construction industry is not limited to natural resources depletion. The volume and management of construction and demolition waste (C&DW) is also a cause of great concern. According to Eurostat [6], 835 million tonnes of C&DW are produced in the European Union per year, representing 36% of the total amount generated and becoming the main waste stream in the EU-27. Estimates put the recycling rate for C&DW at approximately 30%, but most recycling pathways currently in use involve low added-value applications, such as backfilling, which can lead to adverse effects like leaching of pollutants to the surrounding waters. The remaining wastes are either landfilled without receiving any further treatment (30%) or are disposed of in dumps or ditches without control (40%), causing problems related to land occupation and water or air pollution [7,8].

In view of the characteristics of C&DW, the EU identified them as a priority waste stream and has redoubled efforts to encourage their valorisation [9]. The reason behind this lies not only in the alarming environmental footprint associated with C&DW, but also in the enormous potential this waste presents to support the implementation of sustainable development, material efficiency and circular economy policies [10,11,12]. Given the volume and composition of C&DW, the most promising form of recovery for this waste is probably found within the construction sector itself.

In fact, the stony fraction of C&DW (the most abundant, mainly composed of concrete, mortar and ceramic fragments) has been well accepted in the construction industry, primarily in applications as recycled aggregate for concretes or as granular material in road construction or fillings [4,13,14,15,16,17,18,19]. These uses, however, are mainly restricted to the coarse fraction of C&DW (>5 mm). The fines (<5 mm)—consisting predominantly of fine aggregates and cement paste—exhibit some unattractive properties (higher absorption and large proportion of impurities) that limit their reutilisation [20,21]. As a result, this material is often found accumulated outdoors in recycling plants or landfills.

Recent research [22,23,24,25] has explored the viability of recycling the fine C&DW fraction as a supplementary cementitious material (SCM), which arises from the potential cementitious reactivity concealed in the cement paste profusely found in this fraction of C&DW. This provides an industrial outlet for this kind of waste and the consequent decrease in the exploitation of natural resources. Furthermore, as cement is partially replaced by C&DW, the need for its manufacture diminishes. This implies a significant reduction in the material’s environmental footprint, since the cement industry is considered to be one of the main sources of anthropogenic CO_2_ (approximately 7% of global emissions) [26]. The above-mentioned studies have just started paving the way for demonstrating the viability of using the fine fraction of C&DW as supplementary cementitious materials, and further research is still required.

This work addresses the analysis of the behaviour and properties of blended cement pastes containing the fine fraction of recycled concrete (the most abundant type of C&DW [27]) in proportions of 5, 7 and 10%. For this purpose, two classes of fine C&DW samples were selected, in order to take into account the two types of concrete usually manufactured in construction: one of a calcareous nature, originally produced with limestone aggregates (HcG), and a second one obtained from concretes containing siliceous aggregates (HsT). The properties addressed in this paper include the assessment of their potential use as SCM in terms of their reactivity, rheological properties, mechanical performance and their effect on the paste pore network. A life cycle assessment is also carried out in order to quantify the environmental benefits of the valorisation of this fraction of C&DW as SCM.

## 2. Materials and Methods

### 2.1. Materials

**Figure 1:**
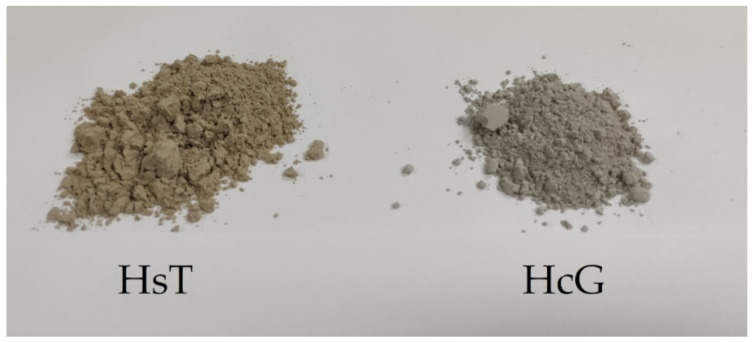
HsT and HcG wastes after processing.

Two different types of waste, resulting from crushing concrete-based C&DW, were chosen for this research. The first one consisted of a siliceous waste (denoted as HsT) derived from concrete originally made with siliceous aggregate, which was supplied by C&DW plants located in the center of Spain (Tecrec, Madrid, Spain). The second one (HcG), was obtained from concrete waste containing calcareous aggregates, and it was provided by plants from northern Spain (Gutram, Basque Country, Spain). The fine waste fraction (<5 mm) used in this work was obtained as a byproduct from the crushing and sieving of the concrete waste carried out in the C&DW treatment plants to obtain coarse granulometric fractions for subsequent industrial use (roads, new concrete, etc.). The fine waste resulting from these processes was subsequently accumulated outdoors in the facilities of the management plants without any industrial use, where it was gathered for this study. The wastes, as received, were heated in an oven at 105 °C for 24 h and then ground in a ball mill to a particle size below 63 μm, similar to that of commercial ordinary Portland cements (OPC). The HsT and HcG wastes after being dried and ground are shown in Figure 1.

The commercial OPC cement employed in this work was a Spanish CEM I 52.5 R cement, supplied by Cementos Lemona, S.A. (Bilbao, Spain). The blended cements were prepared by partially substituting OPC with each of the wastes (HsT and HcG) at 5, 7 and 10% replacement ratios (by weight of binder). The following mineralogical composition was found for OPC by Rietveld analysis: alite (52%), belite (20%), C_3_A (9%), C_4_AF (6%), calcite (4%) and amorphous phase (9%).

### 2.2. Methods

#### 2.2.1. Pozzolanic Activity

The evaluation of the pozzolanic activity of the waste was carried out by means of an accelerated chemical analysis method, consisting of adding 1 g of waste to 75 mL of saturated Ca(OH)_2_ solution and keeping the mixture at 40 °C for the duration of the analysis [28]. At the end of the experiment, the mixture was filtered, and the concentration of calcium remaining in solution was determined by volumetric titration with EDTA. The proportion of lime fixed by the waste was calculated by comparison with the concentration obtained from a blank test.

The reaction kinetics was studied by applying the kinetic-diffusive model described by Villar-Cociña et al. [29,30,31], which allows the determination of the reaction rate constant *K* based on the evolution of lime consumption with time, according to Equation (1):

(1) $$C_{t}=\frac{0.23\;Exp\left(\frac{-3t}{\tau }\right)\cdot \left(-1+Exp(\frac{t}{\tau })\right)\cdot \frac{1}{\tau }}{D_{e}\;r_{s}}+\;\frac{0.23\;Exp\left(-\frac{t}{\tau }\right)\cdot \frac{1}{\tau }}{K\;r_{s}{}^{2}}+C_{corr}$$

where *C_t_* represents the reduction of lime concentration with time (*t*) for the pozzolan/lime system, *D_e_* is the effective diffusion coefficient, τ is a constant that represents the time period in which the radius of the nucleus of the pozzolan is reduced to 37% of the original radius of the average size particle, *r_s_*, and *C_corr_* is a correction term that accounts for the unconsumed lime after the reaction.

#### 2.2.2. Specimen Preparation

The pastes used in this study were prepared at a water/binder ratio equal to 0.5. After mixing, Koch–Steinegger-type specimens were cast in 1 × 1 × 6 cm^3^ prismatic moulds and compacted on a vibration table [32]. The specimens were demoulded after 24 h and cured by immersion in water until they reached the intended age.

#### 2.2.3. Water Demand, Volume Stability and Setting Times

The assessment of water of normal consistency, volume stability and setting times was carried out as described in EN-196-3 [33].

#### 2.2.4. Instrumental Techniques

The flexural strength of the paste specimens was tested on a NETZSCH (Netzsch, Selb, Germany) test frame. Six 1 × 1 × 6 cm^3^ prismatic specimens were tested per type of cement and age. The compressive strength was measured on the specimen fragments resulting from the flexural strength test, using an IBERTEST AUTOTEST 200/10-SW test frame (Ibertest, Madrid, Spain).

The porosity of the pastes was carried out by mercury intrusion porosimetry in a Micromeritics Autopore IV porosimeter (Micromeritics, Norcross, GA, USA). This device operates at pressures that reach 33,000 psi (227.5 MPa), measuring pore diameters between 0.006 and 175 μm.

The elemental composition was studied by X-ray fluorescence on a Philips PW-1404 spectrometer Phillips, Madrid, Spain), fitted with an Sc-Mo X-ray tube.

The particle size distribution was analysed by laser diffraction on a Malvern Mastersizer 3000 analyser (Malvern Panalytical, Madrid, Spain) equipped with red and blue (He-Ne and LED) light sources on dry dispersion mode. The measuring range was from 0.01 to 3500 μm.

The mineralogical analyses were carried out by powder X-ray diffraction (XRD) on a PAN Analytical X’Pert Pro X-ray diffractometer (Malvern Panalytical, Davis, CA, USA) fitted with a Cu anode, operating at 40 mA, 45 kV, and using a divergence slit of 0.5° and 0.5 mm reception slits. The samples were scanned in a 2θ range of 5° to 60°, with a step size of 0.0167 (2θ) at 150 ms/step. Rutile was used as internal standard. Rietveld quantification was performed with Match! v.3 and FullProf suite software (Crystal Impact, Bonn, Germany) and the mineralogical phases were identified using the Crystallography Open Database (COD).

MAS NMR spectra were acquired on a Bruker Avance-400 spectrometer (Bruker, Kontich, Belgium). The operating conditions were: ^27^Al resonance frequency, 104.3 MHz; spinning rate, 10 kHz; pulse width, 2 µs; relaxation delay, 5 s; external standard, Al(H_2_O)_6_⁺^3^; ^29^Si resonance frequency, 79.5 MHz; spinning rate, 10 kHz; pulse width, 5 µs; relaxation delay, 10 s; and external standard, tetramethylsilane (TMS).

SEM/EDX morphological studies were carried out on a FEI Company Inspect scanning electron microscope (Hillsboro, OR, USA) equipped with a W source, an energy dispersive X-ray DX4i analyser and a Si/Li detector.

#### 2.2.5. Environmental Assessment Methodology

In order to evaluate the environmental viability of employing HcG and HsT in new blended cements with partial replacements between 5 and 10%, Life Cycle Assessment (LCA) methodology was selected.

Firstly, the environmental impact of the commercial cement CEM I 52.5R was taken from the European Life Cycle Database (ELCD) [34], and then it was calculated for the HcG and HsT additions. The steps considered for the processing of the HcG and HsT additions were:

- Transport to the valorisation site (20 km).
- Crushing process below 5 mm employing an impact crusher.
- Milling process below 63 µm with a ball mill.
- Transport to the cement factory (30 km).

Material flows and energy consumption to recover the recycled products (HcG and HsT), were estimated using data from commercial crushing and milling equipment. These data are collected in Table 1. The data on the electricity, water and fuel consumption of the background process were taken from the European Life Cycle Database (ELCD) (see Table 2).

Secondly, six scenarios were selected in order to assess the different combinations of blended cement, replacing 5%, 7% and 10% with each of the recycled concrete HcG and HsT:

- S1.1—95% of OPC blended with 5% of HcG.
- S1.2—93% of OPC blended with 7% of HcG.
- S1.3—90% of OPC blended with 10% of HcG.
- S2.1—95% of OPC blended with 5% of HsT.
- S2.2—93% of OPC blended with 7% of HsT.
- S2.3—90% of OPC blended with 10% of HsT.

The following assumptions were considered for the assessment:

- The average European energy values were considered for the energy consumption.
- The energy consumption of the blending process was not considered since it was assumed that it did not add an extra step in the traditional cement production process.
- The distance from the cement plant to the construction sites was supposed to be the same for all the combinations and was not considered in the assessment.

OpenLCA 1.7 software (GreenDelta, Berlin, Germany), CML impact assessment method [38] and the specifications of the EN 15804:2012 standard [39] were used to calculate the environmental impact. The impact categories assessed include: global warming potential (GWP-kg CO_2_ equivalent), ozone depletion (ODP-kg CFC11 equivalent), acidification (AP-kg SO_2_ equivalent), eutrophication (EP-kg (PO_4_)^3−^ eq.), photochemical ozone production (POCP-kg ethylene equivalent), consumption of non-biological resource elements (ADP-E-kg Sb eq.) and consumption of non-biological fossil fuels (ADP- F-MJ). The environmental impact results were normalized according to the normalization factor proposed by CML for the European emission per capita unit for the year 2000 [40].

## 3. Results and Discussion

### 3.1. Characterization of the Fine Fraction of Concrete C&DW Waste

Table 3 displays the elemental composition of the starting materials obtained by X-Ray Fluorescence (XRF). Large variations in composition are observed as a function of the waste studied, finding the greatest differences in SiO_2_, Al_2_O_3_ and CaO. HsT waste is defined by high and low SiO_2_ and CaO content, respectively. Conversely, HcG is dominated by CaO, presenting only moderate amounts of SiO_2_. The values obtained for HsT waste are in agreement with the results determined by other researchers [41]. With respect to loss on ignition (LOI), both HsT and HcG wastes presented high values compared to OPC, mainly due to the decomposition of carbonates and hydrated phases from the original concretes [42].

Regarding the mineralogical composition of the wastes, Table 4 shows the phases identified by X-ray diffraction and their quantification performed by Rietveld refinement. Both materials presented the same crystalline phases, namely mica, quartz, feldspar and calcite. Nevertheless, major quantitative differences were found between them. While quartz (48%) is the main phase in HsT, as expected due to its siliceous nature, calcite (52%) predominates in HcG, as a result of its high calcium content.

**Figure 2:**
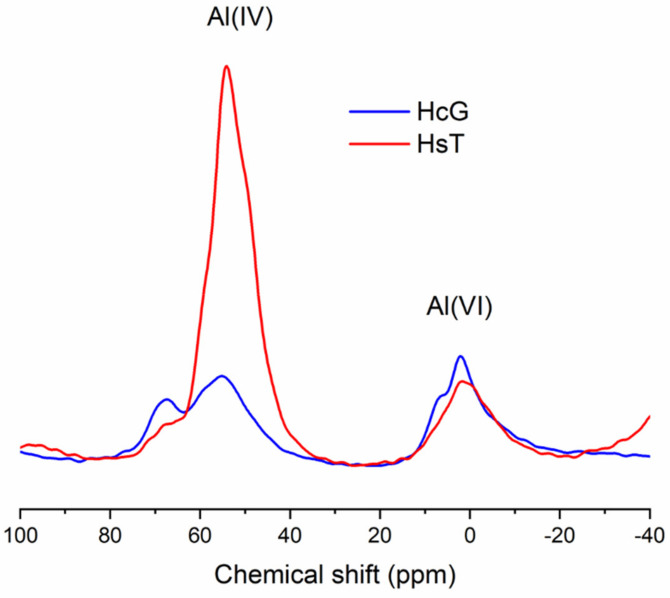
^27^Al NMR MAS spectra for HsT and HcG.

Additionally, both HsT and HcG were measured by ^29^Si and ^27^Al NMR-MAS. The ^29^Si spectrum of HcG revealed a Q^0^ signal at −72.3 ppm, associated with the anhydrous cement phases C_2_S and C_3_S. Q^1^ and Q^2^ peaks were also found at −80 and −86.4 ppm, which are typically related to C-S-H gels and feldspars. An additional signal related to feldspars can be observed at −95 ppm. Lastly, a Q^4^ peak is also detected in the spectrum that corresponds to quartz or reactive silica. The HsT spectrum also features Q^1^, Q^2^, Q^3^ and Q^4^ signals, with Q^2^ and Q^3^ being the most intense peaks, which indicates the presence of silicate structures like C-S-H gels or feldspars, as mentioned above. However, the most relevant information obtained from the HsT spectrum is the absence of Q^0^, indicating the lack of anhydrous phases. On the other hand, the ^27^Al NMR-MAS spectra in both samples showed the presence of aluminium in tetrahedral and octahedral coordination geometries, as indicated by the signals found around −50 and 0 ppm, respectively (Figure 2). While the amount of Al(VI) was similar in both samples, Al(IV) is more abundant in HsT, which is in agreement with the larger proportion of Al_2_O_3_ detected by XRF in this waste [22].

**Figure 3:**
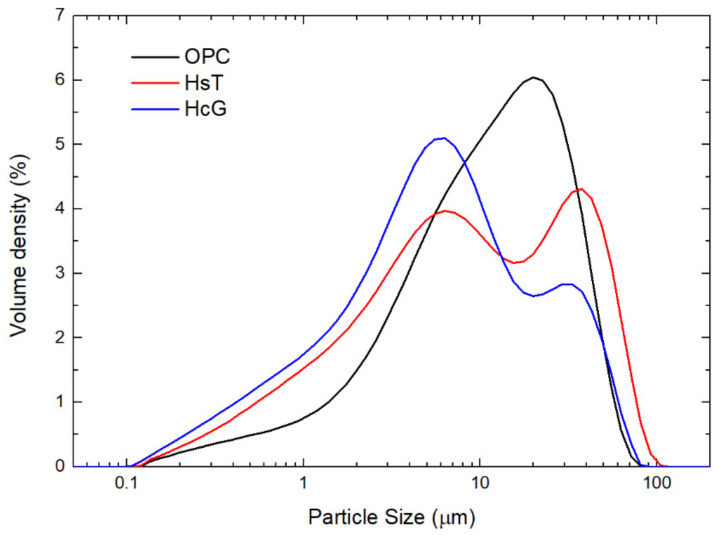
Particle size distribution of the starting materials obtained by laser diffraction.

The particle size distribution of the starting materials, performed by laser diffraction, can be checked in Figure 3. The distribution density curves of the C&DW fine wastes show two maxima, around 32–38 μm and 6–7 μm, respectively, of different intensity, as a consequence of the mineral mixture present in the concrete fine fractions (siliceous and calcareous nature). The intensity of the peaks evidenced the differences in calcite and quartz hardness, inasmuch as the grinding process was similar in both samples. These results were corroborated by the Dx parameters, which are presented in Table 5. OPC presented a slight difference with respect to other samples, presenting a main peak at 20 μm and a shoulder around 8 μm.

### 3.2. Pozzolanic Behaviour of the Fine C&DW Fraction

**Figure 4:**
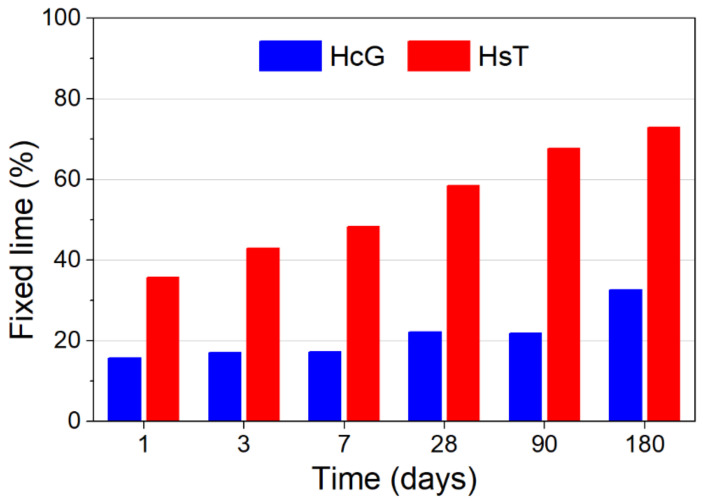
Pozzolanic activity of HcG and HsT wastes in a pozzolan/lime system.

Figure 4 shows the amount of lime fixed over time by HcG and HsT wastes in contact with a lime-saturated solution, which is indicative of the pozzolanic activity of the materials. HcG exhibits low reactivity, which increases slowly over time, consuming only 30% of the available lime after 180 days of exposure. This phenomenon is not unexpected, given that HcG presents a low silica and alumina content and is largely composed of calcite, from which no reaction with lime is foreseeable. Conversely, reactivity is considerably higher for HsT, whose composition is dominated by silica and presents a substantial amount of alumina (approximately 9%). Therefore, HsT is capable of fixing 35% of the available lime at only 1 day of reaction, and this value increases up to 73% after 180 days. While this value is apparently high, it does not compare to conventional pozzolans such as silica fume, metakaolin or fly ash, in which fixed lime values of over 80% can be observed after 90 days of reaction [43,44]. The cause behind the moderate reactivity of HsT is that even though the waste is rich in silica, most of it is found as quartz in the waste, which is considered mostly inert for the timeframes contemplated in this study. Previous works identified small amounts of C-S-H gels and ettringite as reaction products, as well as hydrated calcium aluminates in the case of HcG waste [22].

Table 6 contains the kinetic constants for HcG and HsT obtained by applying Equation (1), as well as the values of other relevant wastes found in the literature, for comparative purposes. As expected, the kinetics of HcG were substantially slower than those of HsT, whose *K* constant was 5–6 times higher. In comparison with the data in the literature, the kinetics of HsT were very similar to those of other construction-related wastes, such as mixed construction and demolition wastes (C&DW) or fired clay discards. However, as expected from its high quartz content, HsT’s reaction rate constant was several orders of magnitude lower than that of other siliceous wastes like vegetable ashes from bamboo leaves or rice husk burning, for instance.

### 3.3. Hydration Products of the Cement Pastes

**Figure 5:**
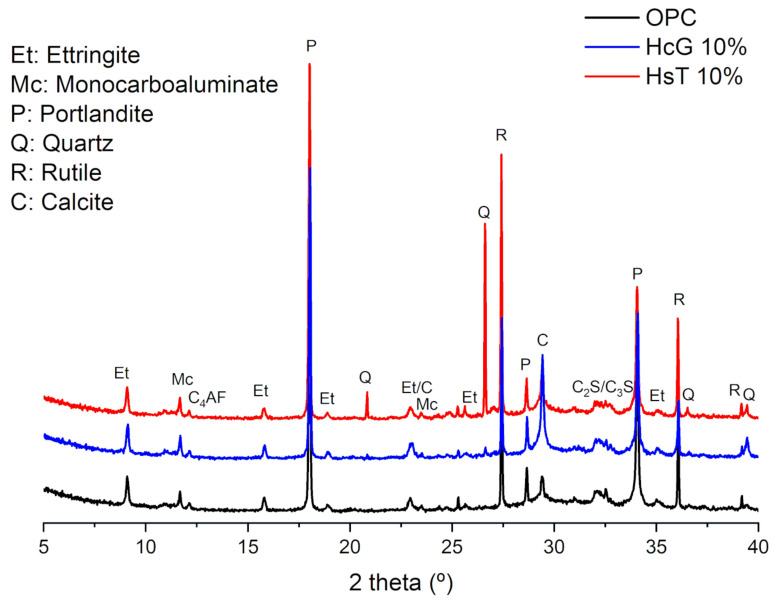
XRD measurements of the samples after 90 days of curing.

The cement pastes were analysed by XRD to determine the mineralogical changes that originated during hydration as a consequence of the addition of HcG and HsT wastes. Pastes hydrated for 90 days with a 10% substitution level were selected for testing, as these were expected to display the greater variations in hydration. According to the results (Figure 5), the partial substitution of OPC by HsT and HcG wastes leads to two main modifications in the diffractograms: (i) the emergence of sharp signals at 26.6° and 20.9° in HsT 10% paste, resulting from the quartz existing in the waste, and (ii) an increase in the calcite signals, which was more prominent in the HcG paste due to its calcareous nature. The remaining signals observed in the diffractograms are analogous for the three types of pastes. Based on them, the crystalline phases portlandite, ettringite and monocarbolauminate could be identified as hydration products, which is in agreement with the results obtained in the study of the pozzolanic reaction in a pozzolan/lime system in previous works [22]. In addition, traces of anhydrous phases from cement can be detected at 12.2° (C_4_AF) and 31.8–33.0° (C_2_S/C_3_S).

**Figure 6:**
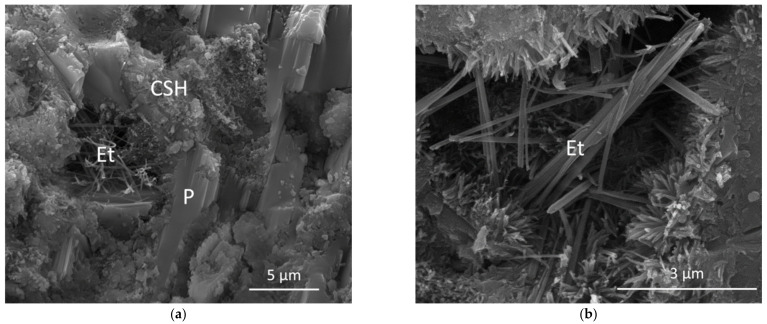
SEM images of (**a**) HsT 7% and (**b**) HcG 10% pastes after 28 days of hydration. CSH: C-S-H gel. Et: Ettringite. P: Portlandite.

The observations of the hydrated pastes carried out by SEM corroborated the results obtained by XRD. Thus, ettringite and portlandite were identified in both HsT and HcG blended pastes. Additionally, the formation of C-S-H gels was clearly detected, which, due to its non-crystalline nature, could not be distinguished by XRD. Some examples of these observations are shown in Figure 6.

**Figure 7:**
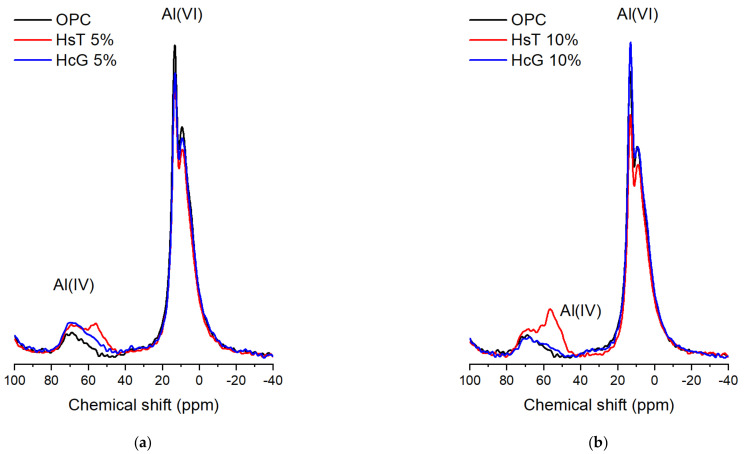
^27^Al NMR MAS spectra of HcG and HsT 90-day pastes, at (**a**) 5% and (**b**) 10% replacement level.

Furthermore, the 90-days hydrated pastes at 5% and 10% replacement levels were analysed by NMR. According to the ^27^Al NMR MAS (Figure 7), all the pastes show signals reflecting the presence of aluminium in both tetrahedral (IV) and octahedral (VI) coordination. The Al (IV) signals can be attributed to aluminium incorporation into C-S-H gels, with the exception of the peak observed at 56 ppm in HsT pastes, which can be assigned to non-reactive tecto- or phyllosilicates from the waste [22]. On the other hand, two overlapping signals, centred at 13 and 9.3 ppm, can be distinguished in the Al (VI) region of the spectra, the former being more intense. These are related to ettringite and AFm phases, the latter possibly corresponding to monocarboaluminate, as found by XRD. No significant differences were found between the blended pastes at 5% and 10% replacement levels.

**Figure 8:**
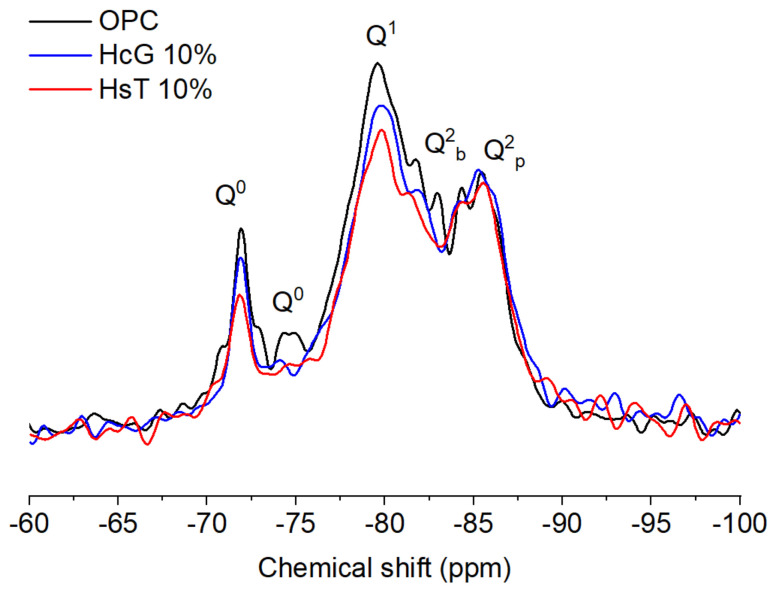
^29^Si NMR MAS spectra of HcG and HsT samples at 10% replacement level.

The ^29^Si NMR MAS spectra (Figure 8) display four different types of peaks, designated as Q^0^, Q^1^, Q^2^_b_ and Q^2^_p_. The Q^0^ signals, corresponding to anhydrous silicates, were reduced upon the addition of the wastes due to cement dilution. This reduction was more pronounced in HsT cements, as this waste does not contain anhydrous phases in its composition, as seen in Section 3.1. In addition, the decrease in the Q^0^ signal might suggest a higher degree of hydration in the blended pastes by filler effect. The remaining signals in the spectra were deconvoluted for the calculation of the Mean Chain Lengths (MCL), which is given in Table 7. According to these, MCL values are higher in the blended pastes. Consequently, the addition of both HsT and HcG leads to a larger connectivity in the silicates and, therefore, to an increase in polymerization and microstructure complexity.

### 3.4. Physical Properties

In order to assess the behaviour of the cement pastes in a fresh state, the physical requirements in the current European regulations were analyzed: normal consistency water (NCW), setting times (IS, FS) and expansion (E) (Table 8). Regarding NCW, a slight decrease was perceived in the pastes containing HcG and HsT with respect to OPC paste. The reductions observed were, however, minimal (less than 3%), so it can be concluded that the addition of HcG and HsT does not significantly influence the consistency of the pastes in the proportions under study. Similarly, the expansion (E) of the pastes is not appreciably affected in any of the pastes. Furthermore, all of them show values well below the threshold established by current European regulations (<10 mm) [48].

**Figure 9:**
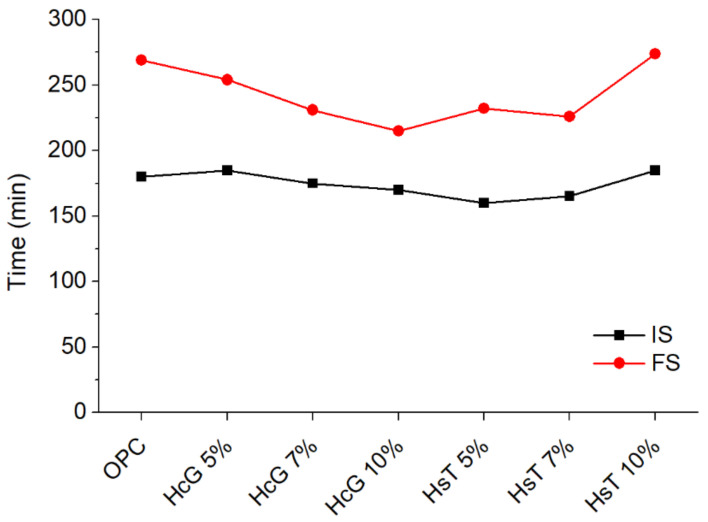
Initial (IS) and final (FS) setting times of the pastes.

In the same line, the initial setting times (Figure 9) were very similar among all the cement pastes. In general, the detected variations lay within the range of error of the technique (±5 min), although some of the blended pastes displayed a minor acceleration with respect to OPC. In any case, they also comply with the limits set by the European regulations for this parameter by a wide margin (≥45–60 min) [48].

Regarding the final setting times (FS threshold not included in the standards), however, there was a clear trend towards acceleration, with the exception of HsT 10% paste. This trend becomes more evident in the pastes containing HcG, in which the acceleration of final setting time is more pronounced as the HcG content increases. This behaviour can be explained by the high calcite content and fineness of the HcG waste, as calcite tends to reduce setting times, particularly at low particle sizes [49,50].

### 3.5. Mechanical Properties of Cement Pastes

**Figure 10:**
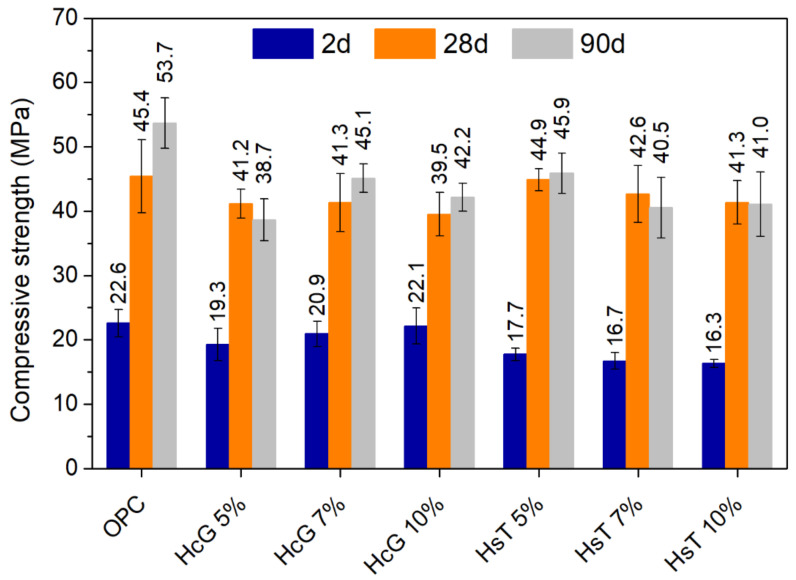
Compressive strength of cement pastes at 2, 28 and 90 days of curing.

**Figure 11:**
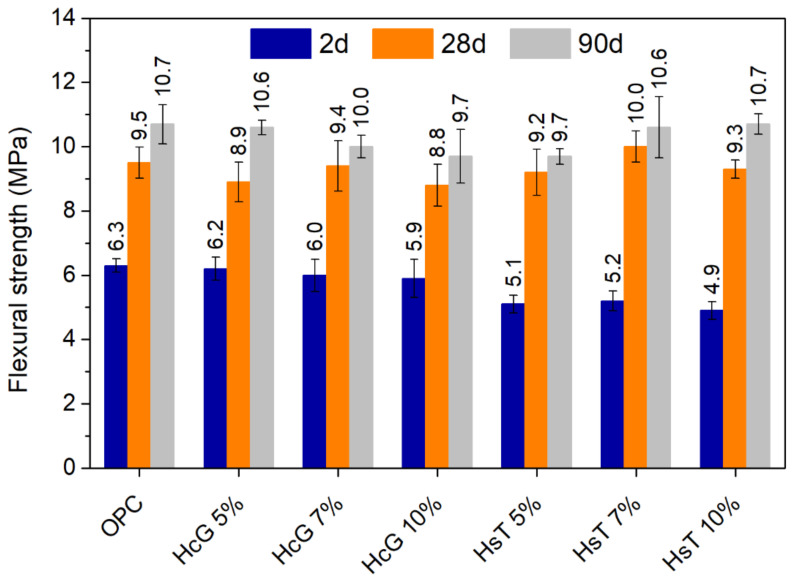
Flexural strength of cement pastes at 2, 28 and 90 days of curing.

Figure 10 and Figure 11 show the compressive and flexural strength, respectively, of the cement pastes at different curing times. After two days of curing, the pastes containing HcG present a mechanical performance that resembles that of OPC (only minimal reductions are found). In contrast, the presence of HsT results in clear strength losses, both in terms of compressive and flexural strength, leading to strength values approximately 20% lower than those of OPC. This is to be expected, as the pozzolanic reaction does not usually take place to a great extent at early ages, as it depends on the release of portlandite from cement hydration. Therefore, the negative effect of cement dilution on mechanical properties usually prevails at young ages. This phenomenon is not applicable in the same way to HcG pastes, as HcG displays weak pozzolanic activity (see Section 3.2), so that their reactivity does not directly rely on the availability of portlandite. Its effect on hydration mainly consists in the formation of carboaluminates (resulting from the high calcite content in the waste), and/or on hydration acceleration due to filler effect [51,52], which may compensate for the dilution effect at early ages, as seen in this case. It must also be highlighted that, as for the filler effect, calcite is considered to be more effective than quartz [40,53].

On the contrary, at 28 days, all pastes were comparable in terms of mechanical strength, if the ranges of variation are taken into account. The reason behind this may be that, as hydration progresses, the pozzolanic reaction in HsT pastes developed and contributed to compensating for the losses in mechanical strength observed at two days of curing. Nonetheless, while OPC compressive strength increased notably from 28 to 90 days of hydration, this effect was not significantly perceived in the blended pastes, which generally present very similar values at both ages. This therefore confirms that the positive effect of the addition of HcG occurs mainly at early ages. In addition, it also reflects the limited pozzolanic potential of HsT owing to its large proportion of inert constituents.

### 3.6. Pore Size Distribution

**Figure 12:**
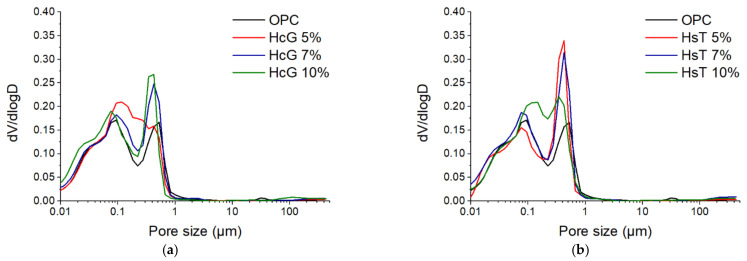
Pore size distribution of (**a**) HcG and (**b**) HsT cement pastes after two days of curing.

The pore size distribution of 2-day-cured specimens is presented in Figure 12. In view of the distribution density curves, it is clear that the greatest differences among them are concentrated in the macropore region (>0.05 µm). For HcG pastes, an increase in the proportion of pores larger than 0.1 µm can be observed with respect to OPC paste. This behaviour is replicated in HsT pastes, although to a greater extent, as the larger pores (around 0.5 µm) become more prominent in these pastes. This is of great relevance, as it is known that the presence of large pores is strongly related to the mechanical properties of cement-based materials [54,55]. Consequently, the pore distributions shown in Figure 12 are in accordance with the mechanical strength losses discussed in the previous section, which were particularly noticeable for HsT pastes.

**Figure 13:**
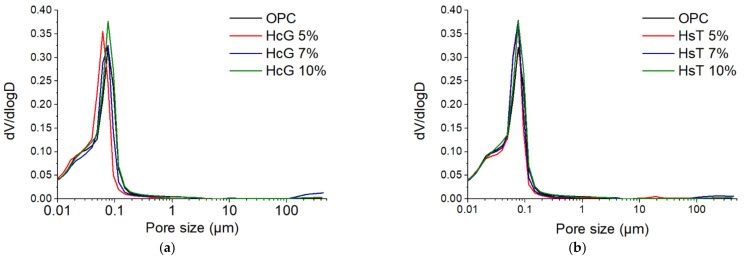
Pore size distribution of (**a**) HcG and (**b**) HsT cement pastes after 28 days of curing.

As a result of the development of hydration and the pozzolanic reaction, the precipitation of primary and secondary hydration products occured, filling the pores and shifting the curves towards smaller sizes. This effect becomes clear in Figure 13, which shows the 28-day pastes pore size distribution curves, these being much more refined than those obtained at 2 days of curing. As a consequence, most pores fell below 0.1 µm, greatly reducing macroporosity. Despite the differences observed at two days of curing, the 28-days pastes presented virtually equivalent pore size distributions. This is also consistent with the mechanical behaviour measured at 28 days, which was not significantly affected by the addition of HcG or HsT. It must also be highlighted that, as a consequence of the similarity in the pore structure, the transport properties (and therefore, the durability) of HcG and HsT pastes can also be expected to be comparable to those of OPC. Nevertheless, specific tests need to be performed to corroborate this.

### 3.7. Environmental Impact Assessment

In a first step, the relative environmental performance of the production of 1 ton of HcG and HsT was assessed and compared with the production of 1 ton of OPC. It can be observed, in Table 9 and Table 10, that the global warming potential (GWP) of HcG and HsT is negligible compared to that of OPC. The obtained benefits in all the impact categories are between 94.4% and 98.7%. This fact is attributable to the simplicity and relative low energy consumption of the recycling process compared to the production of OPC, which is extremely energy-intensive.

In the second step, the environmental impact of the novel blended cements was evaluated in comparison with the OPC. Table 9 and Table 10 show the environmental impact of the partial replacement of up to 10% of the OPC by HcG and HsT. The results revealed reductions of approximately the same order of magnitude as the replacement ratio used, regardless of the original nature of the recycled concrete.

The results can be related to other recently published studies on C&DW concrete sorting technologies [23]. Compared to this other recent study, the reduction of the environmental impact achieved through the route presented in this study was slightly higher than that obtained through other more complex processes.

Thus, the use of the blended cement presented in this work can lead to emission reductions of up to 99 kg CO_2_ eq. per ton of OPC. Considering that the cement industry produced 4.23 million tons of cement in 2018 [56], the use of this novel technique could imply saving up to 0.42 million tons of CO_2_ eq./year.

## 4. Conclusions

The conclusions that can be drawn from this study are as follows:

- Fine concrete C&DW, HcG and HsT exhibit different chemical and mineralogical compositions depending on the nature of the aggregates originally employed (calcareous or siliceous). This fact influences the behaviour and properties of the resulting cement pastes.
- The calcareous C&DW HcG shows weak pozzolanic activity (*K* = 1.18 × 10^−4^) due to its low content of silica and alumina and the predominance of calcite in its composition. HsT waste presents higher pozzolanic reactivity (*K* = 6.68 × 10^−4^), although lower than that of other silica-based pozzolans owing to its high quartz content.
- The main hydration products for the pastes at 90 days are ettringite, portlandite, monocarboaluminate and C-S-H gels, according to XRD and NMR analyses. The blended pastes present longer MCL than those of OPC, suggesting larger microstructure connectivity and polymerization degree.
- The use of HcG and HsT at the substitution levels under study does not lead to significant changes in the fresh properties of the resulting cement pastes, thereby complying with the limits set by European regulations.
- The blended cement pastes prepared with up to 10% of these fine residues show similar compressive strength values to the reference paste at 28 days of curing, but lower at 90 days. This trend is not detected in the flexural strengths, where the values are similar at all curing ages.
- The dilution effect in HsT pastes causes an increase in the proportion of macropores at early ages (2 days), which results in a moderate loss of mechanical strength. This effect is attenuated in HcG pastes due to the greater effectiveness of the filler effect and the formation of carboaluminates. At 28 days, the development of the pozzolanic reaction of HsT compensates for the dilution effect, giving rise to very similar pore distributions and strengths in all the pastes under study.
- The results of the environmental assessment of the studied blended cements revealed reductions of approximately the same order of magnitude as the replacement ratio used, i.e., 9.9% of CO_2_ emissions reduction. By implementing the results of this research, reductions could reach up 0.42 million tons of CO_2_ eq./year in the cement sector.

In view of the studies carried out in this research work, it can be concluded that these fine concrete wastes may be suitable for their valorisation as supplementary cementitious materials, in spite of having been deposited in waste management plants under different adverse atmospheric conditions. Nevertheless, it should be borne in mind that this research area is still in its early stages and that there is a need to advance and generate new knowledge within this field.

## Figures and Tables

**Table 1:** Energy consumption data for recovered HcG and HsT.

Process		Electricity (kWh/t)	Assumptions	Data Source
Crushing (<5 mm)		1.6	Energy consumption of the crushing process was collected from a commercial cone crusher (considering a power of 160 kW and a capacity of 100 t/h) and a scientific article about recycled concrete LCA. The same energy consumption was considered for both streams.	Commercial cone crusher [35], Rosado et al. 2017 [36]
Milling (<63 µm)	HsT	15.5	Energy consumption of the milling process was collected taking into account the bond index of siliceous and limestone minerals.	Tenova Bateman mills (AG/SAG, Rod, Ball Mills) [37]

**Table 2:** LCI dataset employed.

Type of Data	Process	Source
Electricity	Electricity Mix at consumer, AC, <1kV—EU-27	ELCD v3.2
OPC	Portland cement (CEM I), CEMBUREAU production mix, at plant, CEMBUREAU technology mix, EN 197-1	ELCD v3.2
Transport	Lorry transport mix with a 40 t total weight and 27 t max payload	ELCD v3.2

**Table 3:** Chemical composition of the starting materials.

Oxide	OPC	HsT	HcG
SiO_2_	14.22	49.97	9.34
Al_2_O_3_	2.89	8.98	2.88
CaO	69.81	18.65	50.32
Fe_2_O_3_	3.70	2.30	1.20
MgO	0.93	1.37	1.12
SO_3_	3.36	2.53	0.85
Na_2_O	0.33	0.80	0.18
K_2_O	0.76	3.35	0.47
P_2_O_5_	0.14	0.11	0.03
TiO_2_	0.20	0.28	0.14
MnO	0.10	0.04	0.09
LOI	3.22	11.50	33.20

**Table 4:** Rietveld refinement of HsT and HcG (%). Rb: Bragg R factor. X^2^: Rietveld goodness of fit.

Sample	Mica	Feldspar	Calcite	Quartz	Amorphous	Rb	X^2^
HsT	4	8	24	48	16	17.6	7.3
HcG	10	11	52	10	17	23.9	6.9

**Table 5:** Dx values of laser fineness from the wastes.

μm	OPC	HsT	HcG
D (10)	1.99	1.04	0.819
D (50)	11.6	8.42	5.77
D (90)	34.2	44.9	31.9

**Table 6:** Reaction rate constant *K* of HcG and HsT and other wastes from the literature.

Waste	*K* (h^−1^)
HcG	1.18·10^−4^
HsT	6.58·10^−4^
Mixed C&DW [45]	6.19·10^−4^
Fired clay [45]	6.76·10^−4^
Bamboo leaf ash [46]	8.41·10^−1^
Rice husk ash [46]	1.73·10^−2^

**Table 7:** Deconvoluted area values from ^29^Si NMR MAS spectra at 90 days of hydration.

Q^n^	OPC	HsT 5%	HcG 5%	HsT 10%	HcG 10%
Q^0^	14.36	13.63	8.28	7.57	9.07
Q^1^	53.37	50.68	53.31	49.48	53.78
Q^2^_b_	5.81	2.99	4.49	6.42	1.90
Q^2^_p_	26.47	32.70	33.91	36.53	35.26
MCL [47]	3.21	3.41	3.44	3.74	3.38

**Table 8:** Water required for normal consistency (NCW) and volume expansion (E) of the pastes.

Property	OPC	HcG 5%	HcG 7%	HcG 10%	HsT 5%	HsT 7%	HsT 10%
NCW (g)	154	152	151	151	150	152	151
E (mm)	0.5	0.0	0.0	0.0	0.0	0.5	1.0

**Table 9:** Comparison of the environmental impact of OPC and HcG blended cements.

	Ref. OPC (1t)	HcG (1t)		S1.1 95% OPC +5% HcG (1t)		S1.2 93% OPC +7% HcG (1t)		S1.3 90% OPC +10% HcG (1t)	
	Impact	Impact	Impact Difference	Impact	Impact Difference	Impact	Impact Difference	Impact Difference	Impact Difference
GWP [kg CO_2_ eq]	9.03·10^2^	1.15·10^1^	−98.7%	8.59·10^2^	−4.9%	8.41·10^2^	−6.9%	8.14·10^2^	−9.9%
ODP [kg CFC 11 eq]	4.40·10^−5^	2.24·10^−6^	−94.9%	4.19·10^-5^	−4.7%	4.11·10^−5^	−6.6%	3.98·10^−5^	−9.5%
AP [kg SO_2_ eq]	2.21	8.08·10^−2^	−96.3%	2.11	−4.8%	2.06	−6.7%	2.00	−9.6%
EP [kg (PO_4_)^3-^ eq]	2.59·10^−1^	5.18·10^−3^	−98.0%	2.46·10^−1^	−4.9%	2.41·10^−1^	−6.9%	2.33·10^−1^	−9.8%
POCP [kg Ethylene eq]	1.66·10^−1^	4.09·10^−3^	−97.5%	1.58·10^−1^	−4.9%	1.54·10^−1^	−6.8%	1.49·10^−1^	−9.8%
ADP-E [kg Sb eq]	1.43·10^−5^	6.69·10^−7^	−95.3%	1.36·10^−5^	−4.8%	1.33·10^−5^	−6.7%	1.29·10^−5^	−9.5%
ADP-F [MJ]	3.47·10^3^	1.30·10^2^	−96.3%	3.30·10^3^	−4.8%	3.24·10^3^	−6.7%	3.14·10^3^	−9.6%

**Table 10:** Comparison of the environmental impact of OPC and HsT blended cements.

	Ref. OPC (1t)	HsT (1t)		S1.1 95% OPC +5% HsT (1t)		S1.2 93% OPC +7% HsT (1t)		S1.3 90% OPC +10% HsT (1t)	
	Impact	Impact	Impact Difference	Impact	Impact Difference	Impact	Impact Difference	Impact Difference	Impact Difference
GWP [kg CO_2_ eq]	9.03·10^2^	1.24·10^1^	−98.6%	8.59·10^2^	−4.9%	8.41·10^2^	−6.9%	8.14·10^2^	−9.9%
ODP [kg CFC 11 eq]	4.40·10^−5^	2.46·10^−6^	−94.4%	4.19·10^-5^	−4.7%	4.11·10^−5^	−6.6%	3.98·10^−5^	−9.4%
AP [kg SO_2_ eq]	2.21	8.75·10^−2^	−96.0%	2.11	−4.8%	2.07	−6.7%	2.00	−9.6%
EP [kg (PO_4_)^3-^ eq]	2.59·10^−1^	5.43·10^−3^	−97.9%	2.46·10^−1^	−4.9%	2.41·10^−1^	−6.9%	2.33·10^−1^	−9.8%
POCP [kg Ethylene eq]	1.66·10^−1^	4.40·10^−3^	−97.3%	1.58·10^−1^	−4.9%	1.54·10^−1^	−6.8%	1.49·10^−1^	−9.7%
ADP-E [kg Sb eq]	1.43·10^−5^	7.19·10^−7^	−95.0%	1.36·10^−5^	−4.7%	1.33·10^−5^	−6.6%	1.29·10^−5^	−9.5%
ADP-F [MJ]	3.47·10^3^	1.39·10^2^	−96.0%	3.31·10^3^	−4.8%	3.24·10^3^	−6.7%	3.14·10^3^	−9.6%

## Data Availability

Data sharing not applicable.
